# The impact of glenohumeral bone loss on range of motion in patients with anterior shoulder instability

**DOI:** 10.1007/s11845-024-03765-8

**Published:** 2024-08-13

**Authors:** Mohamed Gaafar, Tom R. Doyle, Julia K. Frank, Eoghan T. Hurley, Martin S. Davey, Ailbhe White-Gibson, Sami Khan, Hannan Mullett

**Affiliations:** UPMC Sports Surgery Clinic, Northwood Avenue, Santry, Santry Demesne, Dublin 9 Ireland

**Keywords:** Bankart, Dislocation, Instability, Latarjet, Range of motion, Shoulder

## Abstract

**Background:**

Loss of shoulder range of motion (ROM) is common after surgical management of anterior shoulder instability; however, it remains unclear to what degree this is related to their injury.

**Aim:**

The purpose of this study was to compare passive shoulder ROM in patients with ASI to a normal contralateral shoulder.

**Methods:**

A total of 121 patients undergoing stabilization surgery were prospectively enrolled. Preoperative advanced imaging was used to assess for glenoid bone loss and the presence of off-track Hill-Sachs lesions. Passive ROM was measured in both shoulders while under anaesthesia prior to surgery.

**Results:**

In all directions, there was a significant loss of ROM in shoulders with instability. Regression analysis showed that neither a glenoid bone defect nor greater glenoid bone loss were associated with a loss of ROM in any plane. The presence of a Hill-Sachs lesion was significantly associated with a loss of external rotation, while off-track lesions were associated with a loss of ROM in all planes (*p* < 0.05).

**Conclusion:**

Patients with anterior shoulder instability lost motion in all directions, with a profound loss of external rotation. The presence of a glenoid bone defect nor greater bone loss did not reliably predict a loss of range of motion. A Hill-Sachs lesion was predictive of a loss of external rotation, while an off-track lesion was predictive of a loss of range in all directions.

## Introduction

The glenohumeral joint is the most commonly dislocated joint in the body, often resulting in a loss of function and pain in the shoulder [1]. Anterior shoulder instability (ASI) accounts for 80% of shoulder instability and is better understood than posterior and multidirectional instability [2–4]. Glenohumeral bone loss is associated with increased risk of recurrent shoulder instability, which occurs most frequently in young male contact athletes [5, 6]. Further instability increases the risk of additional intra-articular pathologies such as bone and cartilage loss ultimately leading in the long term to instability arthropathy [7, 8].

The presence of glenoid bone loss as well as the size and location of Hill-Sachs lesions play an important role in the operative management [2–4]. Appropriate preoperative imaging is crucial in order to identify and quantify bony deficiencies [9–11]. In the setting of glenoid bone loss, off-track lesions, or in patients engaged in high-risk activity, a Latarjet or other bone augmentation procedure may be preferred over soft tissue stabilization [8, 12–14]. Several studies report a loss of external rotation of up to 20° after instability procedures [15–17]. However, there is a paucity of literature assessing the range of motion in patients with shoulder instability preoperatively.

The purpose of this study was to evaluate the passive range of motion (ROM) in shoulders with anterior instability and to assess the role of glenohumeral bone loss in reduced range compared with the contralateral side. The hypothesis was that patients with unilateral ASI would have a loss of ROM correlating with greater glenohumeral bone defects.

## Methods

### Patient selection

This study received ethical approval from our institutional IRB. The inclusion criteria for this study were: [1] patients with unilateral shoulder instability, [2] undergoing a primary shoulder stabilisation including arthroscopic or open Bankart repair ± Remplissage or open Latarjet procedure between January 2021 and June 2021 [3] aged 18–39. Patients were excluded if they had contralateral shoulder instability or a rotator cuff tear, a history of ipsilateral shoulder surgery, or a neurological injury.

### Examination technique

Patients under went passive ROM examination of both shoulders while under general anaesthesia in the operating room. ROM including forward flexion, abduction in the coronal plane, external rotation with arm abducted to 90°, and external rotation in adduction was recorded. All measurements were made by a single investigator (MG) using a goniometer prior to the start of the procedure. The mean of three measurements was taken as the final result for ROM in each direction. Anterior instability was graded intraoperatively by the senior author as described by Hawkins et al., with grade 0 (G0) indicating no translation, G1 translation to the glenoid rim, G2 over the glenoid rim, and G3 the head remains over the rim after the examiner stops applying force [18].

### Data collection and clinical outcomes

Data on patient characteristics and pre-operative demographics was collected, including age, sex, and side. All patients underwent magnetic resonance imaging or arthrograms (MRI or MRA). A 3-Tesla magnet was used for all pre-operative imaging (TwinSpeed 8; GE Medical Systems, Milwaukee, WI, USA). A dedicated shoulder surface coil was used, and patients were positioned with the shoulder in a neutral position. T1-weighted fat-saturated coronal, sagittal, and axial images and T2-weighted fat-saturated coronal images were obtained. The MRAs were assessed at the time by a fellowship-trained board-certified musculoskeletal radiologist. Glenoid bone loss was assessed using the best-fit circle method. Furthermore, the glenoid track was evaluated as described by Gyftopolous et al. [19].

### Statistical analysis

All statistical analysis was performed utilizing GraphPad Prism 8.3 (GraphPad, La Jolla, CA). For all continuous and categorical variables, descriptive statistics were calculated. Continuous variables were reported as weighted mean and estimated standard deviation, whereas categorical variables were reported as frequencies with percentages. Categorical variables were analysed using Fisher’s exact or chi-squared test. The independent or paired *t*-test for normally distributed variables or the nonparametric Mann–Whitney *U* test or Wilcoxon signed-rank test was performed to compare continuous variables. A value of *p* < 0.05 was considered to be statistically significant.

## Results

### Patient demographics

Overall, 121 patients undergoing stabilization for ASI were included, with 108 males (89.3%) and a mean age of 23.1 ± 8.1. Overall, 81% were involved in collision sports, 4.1% in contact sports, 11.6% in non-contact sports, and 3.3% were not involved in sports. Anterior instability was graded as G1 in 2.5% of cases, G2 in 66.1%, and G3 in 31.4% of patients. There were 19 patients (15.7%) with a first-time dislocation, while 102 had experienced recurrent instability with a mean 3.3 ± 2.2 dislocations. There were 56 patients with no glenoid bone loss (46.3%), the mean glenoid bone loss was 8% ± 9% (range 0–27%), 87.6% had a Hill-Sachs lesion, while 30.6% had an off-track Hill-Sachs lesions. Surgery was performed a mean of 8.3 ± 5.4 weeks after the most recent dislocation event (0.5–36 weeks).

### Range of motion

Overall, the ROM in all planes was significantly lower in the shoulder with ASI than in the contralateral shoulder, with forward flexion of 177° ± 11° vs 179° ± 1°(*p* < 0.01), abduction of 171° ± 8° vs 179° ± 1° (*p* < 0.01), external rotation measured in adduction of 56° ± 13° vs 89° ± 4° (*p* < 0.01) and in abduction 67° ± 11° vs 94° ± 7°(*p* < 0.01). This is shown in Table 1. There were no significant differences in ROM between right vs left, males vs females, primary vs recurrent dislocators (*p* > 0.05 for all).

**Table 1:** Range of motion vs contralateral side

	Unstable shoulder	Contralateral shoulder	Difference	*p* value
Forward flexion	177° ± 11°	179° ± 1°	− 2° ± 11°	0.005
Abduction	171° ± 8°	179° ± 1°	− 8° ± 8°	< 0.001
External rotation^1^	56° ± 13°	89° ± 4°	− 33° ± 13°	< 0.001
External rotation^2^	67° ± 11°	94° ± 7°	− 27° ± 13°	< 0.001

^1^Measured in adduction

^2^Measured in 90° abduction

### Relationship between instability and glenohumeral bone lesions

Linear and binomial logistic regressions were used to assess for factors related to bony lesions. The number of preoperative dislocations was associated with the presence of a glenoid bone defect (*p* = 0.009), greater glenoid bone loss (*p* < 0.001), a Hill-Sachs lesions (*p* = 0.044) and an off-track Hill-Sachs lesion (*p* = 0.013). G3 intraoperative instability was associated with greater percentage glenoid bone loss when compared to G2 (*p* = 0.026) and G1 (*p* = 0.002).

### Relationship between glenohumeral bone lesions and range of motion

When comparing patients with a glenoid bone defect vs those with no glenoid bone loss, there was no clinical or statistically significant difference in the loss of ROM between the groups as shown in Table 2. The Hill-Sachs lesions cohort saw a greater loss in external rotation in adduction (− 34° ± 12° vs − 24° ± 15°, *p* = 0.003) and in abduction (− 28° ± 11° vs − 20° ± 11°, *p* 0.012). The presence of an off-track Hill-Sachs lesion was associated with a significant loss of range in all planes as shown in Table 2.

**Table 2:** The effect of glenohumeral bone lesions on the loss of range of motion compared to the uninjured contralateral side

	**No lesion**	**Lesion**	***p* value**
**Glenoid bone defect**			
Flexion	− 2° ± 9°	− 4° ± 5°	0.217
Abduction	− 9° ± 9°	− 8° ± 7°	0.601
ER1	− 33° ± 13°	− 33° ± 13°	0.878
ER2	− 28° ± 11°	− 26° ± 11°	0.388
**Hill-Sachs**			
Flexion	− 4° ± 7°	− 3° ± 11°	0.676
Abduction	− 6° ± 7°	− 9° ± 8°	0.155
ER1	− 24° ± 15°	− 34° ± 12°	**0.003**
ER2	− 20° ± 11°	− 28° ± 11°	**0.012**
**Off-track Hill-Sachs**			
Flexion	− 2° ± 12°	− 6° ± 6°	**0.038**
Abduction	− 7° ± 7°	− 13° ± 8°	** < 0.001**
ER1	− 29° ± 12°	− 41° ± 12°	** < 0.001**
ER2	− 25° ± 11°	− 31° ± 11°	**0.007**

*ER1* external rotation in adduction, *ER2* external rotation in abduction

Using multi-linear regression, neither a glenoid bone defect nor greater percentage glenoid bone loss were associated with a significant loss of ROM in any plane, as shown in Table 3. The presence of a Hill-Sachs lesion was significantly associated with loss of external rotation measured in adduction (*p* = 0.007) and abduction (*p* = 0.022). While the presence of an off-track lesion was significantly associated with a loss of ROM in all planes (*p* < 0.05 for all). There was no significant relationship between the number of weeks from dislocation to surgery and loss of ROM for flexion (*p* = 0.734), abduction (*p* = 0.525), external rotation in adduction (*p* = 0.114), or abduction (*p* = 0.572).

**Table 3:** Glenohumeral bone loss and relationship to loss of range of motion

Variable	Estimate	95% CI	*p* value
Forward flexion			
Bony Bankart	− 3.426	− 10.326 to − 0.984	0.372
Glenoid bone loss	− 0.114	− 0.436 to 0.117	0.330
HSL	2.070	− 4.223 to 8.362	0.516
Off-track HSL	− 2.949	− 9.302 to − 0.596	**0.026**
Abduction			
Bony Bankart	− 0.757	− 10.040 to 8.527	0.872
Glenoid bone loss	− 0.012	− 0.305 to 0.270	0.879
HSL	− 1.593	− 6.050 to 2.865	0.480
Off-track HSL	− 5.237	− 8.324 to − 2.149	**0.001**
External rotation arm at side			
Bony Bankart	− 2.046	− 9.619 to 5.526	0.594
Glenoid bone loss	− 0.027	− 0.278 to 0.223	0.829
HSL	− 7.889	− 14.576 to − 1.203	**0.021**
Off-track HSL	− 10.199	− 14.949 to − 5.430	** < 0.001**
External rotation in abduction			
Bony Bankart	0.993	− 5.832 to 7.818	0.774
Glenoid bone loss	0.039	− 0.187 to 0.267	0.729
HSL	− 7.250	− 13.420 to − 1.080	**0.022**
Off-track HSL	− 4.794	− 9.063 to − 0.526	**0.028**

Bold indicates statistically significant

*HSL* Hill-Sach’s Lesion, *CI* confidence interval

## Discussion

The most important finding of this study was that patients with anterior shoulder instability lost passive range of motion in all directions, with a particular loss of external rotation observed. Neither the presence of a glenoid bone defect nor greater glenoid bone loss was significantly associated with a loss of range of motion in any plane. There was an association between the presence of a Hill-Sachs lesion and loss of external rotation measured in adduction and abduction, while an off-track lesion was associated with loss of range in all planes of motion. Recurrent dislocation was associated with greater glenohumeral defects which in turn predicted loss of range of motion, highlighting the negative impact of recurrent instability on shoulder function. Concerns regarding restricted range of motion after shoulder stabilization are commonly raised [20, 21]; however, restricted range appears to be at least partly mediated by the original injury to the shoulder.

Loss of ROM is an important consideration in patients of all ages with shoulder instability. In younger athletes for instance, it is important in returning to pre-morbid function and athletic activities, whereas in older patients with concomitant pathologies such as rotator cuff tears, this may contribute to loss of functional motion [3, 22]. Evaluation of ROM is also an important part of post-operative testing in clearing athletes to return to play [23]. However, in evaluating post-operative recovery of ROM, it is important to understand that pre-operative loss of ROM can occur with glenohumeral bone defects effecting the patients baseline range. Additionally, in overhead athletes, this may be a limiting factor in being able to successfully return to play [14, 24–27]. Therefore, it is important to counsel patients that the risk of bony defects increases with further instability events and both on- and off-track Hill-Sachs lesions are associated with restricted ROM; these factors should be considered in the management of first-time shoulder dislocation, and early surgical intervention may be considered depending on the patient’s risk profile and functional demands.

The greatest deficits were observed in external rotation, with patients losing a third of their range. This can be problematic for throwers and those who require overhead function [20, 23, 27]. The presence of a Hill-Sachs lesion and an off-track lesion was significantly associated with restrictions of external rotation. Similarly, an off-track lesion was associated with a loss of range of flexion and abduction. The glenoid track (GT) is the zone of contact between the humeral head and the glenoid, while 83% of the glenoid diameter is commonly accepted as the GT this figure is the mean result from the original study [28]. Yamamoto and Itoi have previously shown that the GT width is narrowed in greater abduction and external rotation as the lateral side of the humeral head contacts the glenoid [29–31]. Despite being examined under anaesthesia, it appears that patients with off-track lesions in our study may have developed a restricted ROM to avoid engaging their Hill-Sach’s lesion and dislocating as the GT narrows at terminal range. Off-track lesions have previously been identified as the greatest risk factor for patients experiencing apprehension in all ranges [32]. Apprehension has classically been defined to involve an involuntary response [33] and is a complex process which is mediated at a neuronal level both centrally and peripherally, with alterations in proprioception, brain activity, and reflex arcs [34]. Further research will be required to explore the effect of glenohumeral bone defects on active ROM and to explore the role of apprehension which commonly persists after stabilization even in the absence of recurrent dislocation [34].

### Limitations

It may be considered a limitation that measurements were made by a single examiner who was not blinded to the patient’s history. Additionally, the shoulder ROM was evaluated while patients were under anaesthesia and active ROM was not compared which may limit the clinical application of these findings. The timing from injury to surgery was not standardized, although it was shown not to influence the loss of ROM. Glenohumeral bone loss was measured via MRI which while valid may be less accurate than measurement with CT, although this reflects our clinical practice in this population [9].

### Conclusions

Patients with anterior shoulder instability lost motion in all directions, with a profound loss of external rotation. The presence of a glenoid bone defect nor greater bone loss did not reliably predict a loss of range of motion. A Hill-Sachs lesion was predictive of a loss of external rotation, while an off-track lesion was predictive of a loss of range in all directions.
